# A case of death of patient with ovarian fibroma combined with Meigs Syndrome and literature review

**DOI:** 10.1186/s13000-022-01258-9

**Published:** 2022-10-17

**Authors:** Na Tan, Kai-yuan Jin, Xiao-rong Yang, Cheng-fang Li, Jin Yao, Hong Zheng

**Affiliations:** Department of Pathology, Affiliated Hospital of Zunyi Medical University, Zunyi, Guizhou China

**Keywords:** Ovarian fibroma, Meigs Syndrome

## Abstract

Ovarian fibroma is the most common benign pure stromal tumor. It has no specific clinical manifestation, most of which are pelvic or adnexal masses. 10-15% of cases with hydrothorax or ascites, after tumor resection, hydrothorax and ascites disappear, known as Meigs Syndrome. The elevated level of CA125 in a few patients was easily misdiagnosed as ovarian malignant tumor. A case of bilateral Ovarian fibroma associated with Meigs Syndrome is reported and the literature is reviewed in order to improve the understanding of the changes and avoid misdiagnosis.

## Case report

**Fig. 1:**
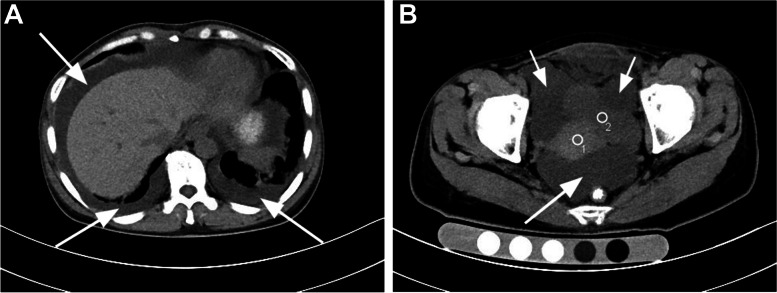
A and B, CT coronal images through the 10th thoracic vertebrae and the pelvis demonstrate there were multiple irregular cystic and solid masses in bilateral adnexa (White small arrow). A large amount of effusion (white big arrowhead) is present within the bilateral thoracic cavity and pelvic and abdominal cavity. the uterus (circle 1), the bladder (circle 2)

A 46-year-old female was presented with recurrent abdominal distension and pain for 4 years. 4 years ago, the patient complained of epigastric distention and pain with no apparent cause as well as nausea and vomiting. After an outpatient examination at another hospital, duodenal stenosis was suspected, and the symptoms improved without hospitalization after conservative symptomatic treatment. The above symptoms reappeared and worsened 1 month ago, with vomiting evident after eating and abdominal distension and discomfort. Weight loss of approximately 5 kg over one month, and during admission, BMI (Body mass index) was 21.37. The differential diagnosis: duodenal stenosis, pyloric obstruction? ovarian cancer? Observations by ultrasound: bilateral adnexal mass, pelvic and peritoneal effusion. Plain and enhanced CT scan of the abdomen (see Fig. 1): there were multiple irregular cystic and solid masses in bilateral adnexa, shows left ovary 7 cm x 6 cm x 6 cm and right ovary 8 cm x 7 cm x 7 cm, considered neoplastic lesions. It should think about the possibility of ovarian cancer. Moreover, a large amount of effusion was found in the bilateral thoracic cavity and pelvic and abdominal cavity. There was effusion in abdominal soft tissue. Blood investigation: Female tumor-associated antigen CA19-9 41.10 U/mL and CA125 56.5 U/mL (normally < 35 U/mL), neutrophils 2.45 × 109/L, while other parameters are normal. Biochemical tests of ascites: total proteins 42.3 g/L, RIVALTA test (+). The clinical diagnosis: causes of abdominal distension: metastasis and invasion of ovarian cancer? pyloric obstruction? multiple abdominal metastases? Before surgery, MDT meetings were held among the departments of gastrointestinal surgery, obstetrics and gynaecology, and anesthesia. The operation was recommended due to the high likelihood of malignancy and lack of obvious contraindications to surgery. Laparoscopic surgery was performed collaboratively by gastroenterologists and gynecologists. Intraoperative observation: there exited adhesion with a hard texture between the middle segment of the greater omentum and lower margin of the right hepatic lobe and between the duodenal bulb and pyloric part. A large amount of pale blood fluid could be found under the left subphrenic space, right liver lobe and pelvic cavity. The bilateral ovaries showed nodular and beaded changes, with multiple nodules. The nodules had different sizes, with slightly hard quality and acceptable mobility. Intraoperative frozen sections: spindle cell tumor. It was considered the diagnosis of sex cord-stromal tumor. Subtotal gastrectomy and gastrojejunostomy + distal duodenal exclusion + complex enterolysis + bilateral accessory and hysterectomy were performed. On the 8-th postoperative day, frequent vomiting of green gastric juice began, and gastrointestinal decompression was used to treat it. On the 13-th postoperative day, recurrent fever up to 40.1 °C began, and a CT scan of the chest revealed increased pleural effusion. On the 14-th postoperative day, blood was cultured, confirming the presence of G coccus (+), and the antibiotic was then changed to Cefoperazone-tazobactam. In the latter half of the day, the blood culture was done again, which confirmed the presence of Enterococcus gallinarum (+). Eventually, the antibiotic was changed to Moxifloxacin and Amoxicillin, and Flucloxacillin. Due to infectious anemia and coagulation abnormalities, a total of 1200 ml of blood was continuously transfused on the 16th postoperative day, including 4 u of suspended red blood cells and 400 ml fresh frozen blood plasma. A 24-hour urine output of 200 ml was recorded on the 17th postoperative day, and the patient was diagnosed with secondary coagulation abnormalities, sepsis, and multi-organ failure. Due to the continuous deterioration of the high cost of ICU treatment, the patient’s family lost confidence in continuing treatment and requested to be discharged. A follow-up phone call confirmed that the patient passed away at home three days after being discharged leaving from the hospital.

## Pathological examination

**Fig. 2:**
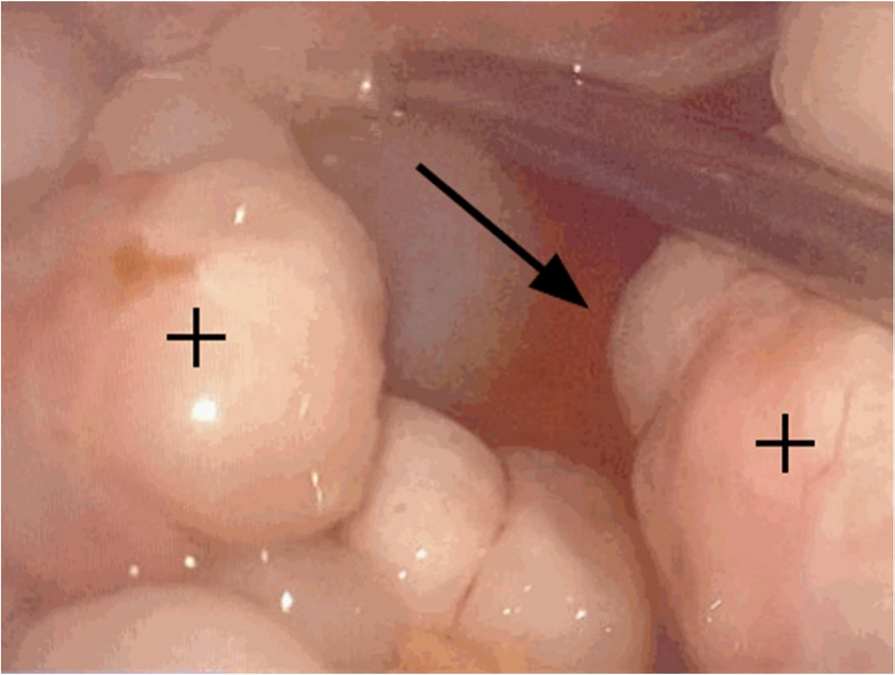
Laparoscopic Intraoperative photograph, can be found bilateral ovarian fibroma (black cross) and ascites (black arrow)

Observation by the naked eye: the sizes of bilateral ovaries were 8 × 6 × 4 cm^3^ and 7 × 5 × 2 cm^3^, respectively. The surface of the ovaries was smooth with multinodular and beaded changes. The size of the nodules varied, with the largest diameter of 3 cm (see Fig. 2). The surface section was off-white, with moderate hardness and solid structure. The ovaries were cystic locally and the thickness of the cyst wall was 0.1 cm with colloidal substances inside. There was no bleeding or necrosis.

**Fig. 3:**
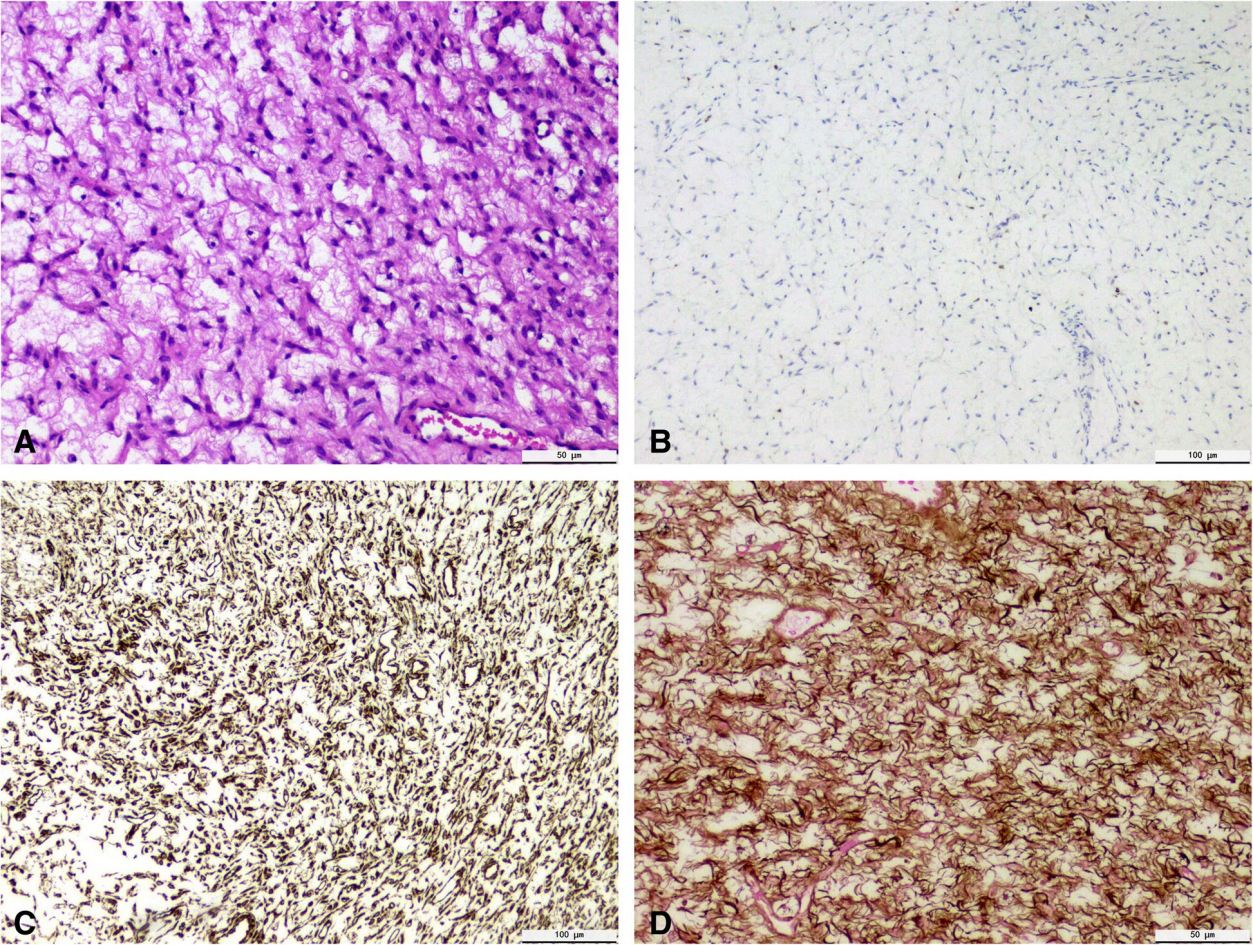
A Histology: showed a large number of spindle cells or ovoid cells, Tumor cell nucleus is long fusiform. There is an obvious edema between cells (400×). B Immunohistochemistry: α-inhibin weakly positive (100×). C Immunohistochemistry: Vimentin positive (100×). D Immunohistochemistry: Reticular fiber positive (400×)

Microscopic examination (see Fig. 3): the ovarian structure disappeared. A large number of spindle cells or oval cells are arranged in feathery, braided, or whirl-like, occasionally storiform. The nuclei of tumor cells were long fusiform without mitotic figures. There was no lipid in the cytoplasm. The intercellular edema was prominent without mucoid degeneration, hyaline degeneration, calcification and ossification. The results of immunohistochemistry showed that tumor cells: Vimentin, WT-1, Calretinin (partial expression), Ki-67 (3% positive rate), α-inhibin (+), reticular fiber dyeing (+).

Pathological examination: ovarian fibroma.

## Discussion

According to the definition of female reproductive system diseases proposed by WHO in 2020, ovarian fibroma is a benign pure stromal tumor composed of the spindle to oval fibroblasts like cells that produce collagen. Ovarian fibroma is the most common sex cord-stromal tumor, accounting for 4% of all ovarian tumors [1]. It can occur at any age, but it is most common in middle age (average 48 years old). It seldom occurs before 30 years old and has also been reported in infants of 7 months old [2]. Most of the tumors are unilateral and rarely bilateral. The tumor’s average size is 8 cm, about 1/3 of the tumors can be less than 3 cm. There was no specific clinical manifestation and most of them show pelvic or adnexal masses [3].

About 10-15% of ovarian fibroma can be combined with pleural fluids or ascites, described as Meigs syndrome. It was reported by Meigs and Cassa in 1937. In 1954, Meigs Syndrome was defined as a benign ovarian fibroma or fibroid tumor with pleural fluids and ascites. The pleural fluids and ascites disappeared after tumor resection, and ascites recurred in tumors with a diameter > 10 cm. The possible cause of pleural fluids and ascites is that the fluid within tumors infiltrates the abdominal cavity through the serosal surface. It then enters into the unilateral or bilateral pleural cavity through the lymphatic vessels or the communication between the pleural cavity and the abdominal cavity [4].

There are no specific markers for accurate diagnosis of ovarian fibroids/fibroids before surgery [5]. CA125 level in serum increased in a few patients. Some studies suggested that increased CA125 level in serum might be related to some biochemical factors, such as elevated intraperitoneal pressure caused by mechanical stimulation of ascites to the peritoneum, or peritoneal mesothelial cells, which might be misdiagnosed as endometriosis or malignant ovarian tumor [6]. In this case, the patient had an elevated CA125 and ascites before the operation, which was easily misdiagnosed as a malignant ovarian tumor.

According to the proportion of fibroblasts and collagen fibers, ovarian fibroma can be divided into cellular and fibrous types. Cellular fibroma is associated with ovarian rupture and extraovarian adhesions and has a risk of local recurrence. Besides, fibroma has some particular subtypes. Fibroma with a small amount of sex cord components refers to less than 10% tubules with different differentiation degrees or incomplete tubular structure of immature supporting cells in fibroma. Moreover, there are abundant tumor cells arranged closely with unclear cytoplasmic boundaries and fewer collagen components. The nuclear chromatin increases and is in round or oval with or without mild atypia, and mitotic rates are ≥ 1–3/10 HPFs. In this case, it can be called cell-rich fibroma. Cell-rich fibroma commonly presents a benign clinical course but has low malignant potential and occasional local recurrence [7–9].

It should be differentiated from the following diseases:

1. Thecoma: thecoma is another kind of pure stromal tumor. However, fibroma and thecaoma have overlapping morphological features. Some cases that are difficult to distinguish can be called fibrothecoma or follicular fibroma. Microscopically, several relatively isolated theca cells rich in lipid between fat spindle-shaped fibroblasts or fibroblasts, showing clump distribution. However, if the patient has no steroid hormone changes and the composition of theca cells in tumors is less than 10% with negative immunohistochemical inhibin, it is not recommended to be diagnosed as fibrothecoma but directly diagnosed as a fibroma. Theca cells in this patient are not rich in lipid and have loose fibrous stroma with prominent edema.
2. Massive edema of the ovary: when fibroma shows obvious edema, it should be differentiated from this disease. Most of the patients with massive edema of the ovary are younger than 30 years old, and abdominal pain is the main clinical symptom. Ovaries have the normal structure, and the whole ovary is diffuse with edema, and sometimes luteinized cells can be found in the stroma. The patient, in this case, does not have ovaries with the normal structure.
3. Fibrosarcoma: the tumor cells with moderate to severe atypia are very abundant, arranged in braided shape, with eosinophilic cytoplasm and unclear boundaries. The nuclei are in round or fusiform shape, with abundant chromatin, prominent nucleolus. Mitotic images are common, with an average of > 4/10 HPFs. There usually are bleeding and necrosis complications. The transitional zone between fibroma and fibrosarcoma can be found in a few tumors. Fibrosarcoma has a poor prognosis and a few patients had been reported to survive more than 5 years.
4. The identification of Meigs Syndrome:

Pseudo Meigs Syndrome: in addition to ovarian fibroma, ascites and pleural fluids occur in patients with other benign or malignant tumors in the pelvis or abdomen [10].

Pseudo-Pseudo Meigs Syndrome: it is also known as Tajalma Syndrome. It can be found in patients with systemic lupus erythematosus accompanied by ascites, pleural fluids, and elevated CA125 level in serum [11].

The patient had ovarian fibroma with pleural fluids and ascites, accompanied by slightly elevated CA125. There were no other tumors or systemic lupus erythematosus. After bilateral accessory and hysterectomy, the pleural fluids and ascites disappeared temporarily, consistent with Meigs Syndrome. The subsequent recurrence of pleural fluids and ascites was due to severe infection rather than the disease itself.

Based on the ultrasound imaging, the typical ovarian fibroma can be manifested as a well-defined hypoechoic mass in the accessories, acoustic attenuation and minimal Doppler flow signal, as well as pleural fluids or ascites. However, it can be easily misdiagnosed as a malignant ovarian tumor due to its lack of understanding. However, it should also be noted that fibroma can spread out of the ovary and adhere to the surrounding tissues in sporadic cases. In this case, the patients have a poor prognosis and infection complication with increased recurrence risk. The recurrence time can be more than 10 years after the operation. An improved understanding of the disease is helpful to avoid misdiagnosis and missed diagnosis.

We believe the patient died as a result of an uncontrollable postoperative infection and the refusual from patient’s family to pursue further treatment. We presume that similar tragic situations could be avoided if disease awareness was increased, early and accurate diagnosis was achieved, and less invasive treatment options were chosen to reduce complications.

## Data Availability

As a case report, all data generated or analyzed are included in this article.

## Abbreviations

BMI: Body mass index
WT-1: Wilms tumor- 1 protein
Ki-67: Marker of proliferation Ki-67
HPFs: High power fields
